# Impact of treatment strategies incorporating sacubitril/valsartan on achievement of guideline-recommended blood pressure targets and representative safety outcomes

**DOI:** 10.1038/s41440-025-02537-w

**Published:** 2026-01-14

**Authors:** Tomohiro Katsuya, Fumiko Nakatsu, Shunsuke Eguchi, Yumiko Nakamura, Miyuki Matsukawa, Kazuma Iekushi, Shinya Hiramitsu

**Affiliations:** 1 Katsuya Clinic, Amagasaki, Japan; 2 Medical Affairs, Novartis Pharma K.K., Tokyo, Japan; 3 Medical Affairs, Otsuka Pharmaceutical Co., Ltd., Tokyo, Japan; 4 Hiramitsu Heart Clinic, Nagoya, Japan

**Keywords:** Hypertension, Sacubitril/valsartan, Real-world evidence, Japan, Implemental hypertension

## Abstract

This real-world, non-interventional, retrospective cohort study evaluated the achievement rate of guideline-recommended target blood pressure (BP) and representative safety profile of the treatment incorporating sacubitril/valsartan (Sac/Val) in Japanese patients with essential hypertension. Data were collected from electronic health records from ~4700 clinics across Japan, covering ~11.4% of the nationwide population. Of the 1405 eligible patients, 1247 were included in the effectiveness analysis. The primary endpoint investigated the proportion of patients achieving the Japanese Society of Hypertension 2019–recommended antihypertensive goals within 8 weeks of initial Sac/Val administration (index date). Secondary endpoints included description of baseline characteristics and their relative contribution to BP goal attainment, description of prescription patterns, and safety. A total of 29.8% of patients achieved individual estimated BP goals, with significant mean reductions in systolic and diastolic BPs (−15.6 mmHg and −6.1 mmHg, respectively, *p* < 0.0001). Patients aged ≥75 years, those with cerebrovascular disease, and those classified as Grade I hypertension were more likely to meet BP goals. Among patients with BP reduction of ≥10 mmHg, the most common prescription pattern at index date was a combination of calcium channel blocker (CCB) and Sac/Val, and a majority switched from CCB and angiotensin receptor blocker combination or were on CCB monotherapy. The most common signs of adverse events were hypotension and diuresis-related events, particularly during summer. The discontinuation rates following these signs were 1.0% and 0.8%. This real-world study demonstrated the clinical utility and representative safety profile of treatments involving Sac/Val in Japanese patients with essential hypertension.

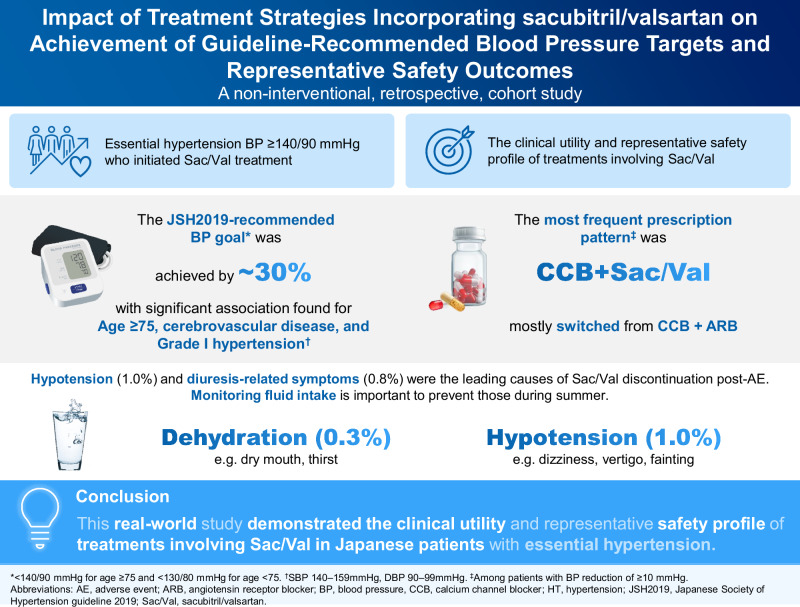

## Introduction

Hypertension, a chronic condition in which blood pressure (BP) levels remain above normal, affects over 43 million people in Japan [1], with higher prevalence among men 50 years and women 60 years of age [2]. The healthcare costs related to cardiovascular diseases, including hypertension, accounted for ~20% of the total medical expenditure in 2017, equating to 6.1 trillion Japanese yen or US$52.1 billion [3]. Hypertension also contributes to the onset of cardiovascular diseases, stroke, and premature death [4].

The Japanese Society of Hypertension (JSH) 2019 guideline (JSH2019) recommend a BP target of <130/80 mmHg for most adults and <140/90 mmHg for elderly patients aged ≥75 years [5, 6]. There are significant gaps in hypertension care in Japan, with 33% of hypertensive patients being unaware of their condition, only 56% receiving treatment, and only 27% reporting well-controlled BP [7].

Sacubitril/valsartan (Sac/Val; LCZ696) is a first-in-class angiotensin receptor–neprilysin inhibitor (ARNI) combination approved in many countries globally, including Japan, for patients with chronic heart failure [8, 9]. In Japan, Sac/Val 100, 200, and 400 mg doses have also been approved for hypertension and are used once daily. Evidence indicates that the antihypertensive effect of Sac/Val is greater than that of angiotensin II receptor blockers (ARBs) [8, 10–13]. Sac/Val was effective in Japanese patients with mild to moderate essential hypertension, providing better control of BP and achieving target BP goals more effectively [8, 14].

The 2021 Japanese heart failure treatment guidelines recommend switching from angiotensin-converting enzyme inhibitors (ACEi)/ARBs to Sac/Val for patients who remain symptomatic despite treatment [15]. However, there are currently no specific recommendations regarding the optimal regimen for Sac/Val administration in hypertensive patients. Combination therapy is generally advised for individuals presenting with elevated baseline BP or for those who fail to achieve target BP goals (<130/80 mmHg for most adults and <140/90 mmHg for elderly patients aged ≥75 years) when treated with monotherapy [6].

The current study investigated the effectiveness and safety of Sac/Val in BP control among Japanese patients with hypertension in a real-world setting through database analysis of electronic health records.

Point of view

- Clinical relevance
  This real-world study highlights the clinical utility and representative safety profile of treatments involving Sac/Val in Japanese patients with essential hypertension.
- Future direction
  In light of the recent JSH guideline update, which sets a uniform BP target of <130/80 mmHg for all patients, future studies should determine whether Sac/Val can facilitate safe attainment of these stricter goals and establish the optimal treatment regimen.
- Consideration for the Asian population
  Hypertension risk increases with age, and Asia’s rapidly aging population suggests a growing predominance of elderly hypertensive patients. Because older individuals exhibit greater BP changes in response to ambient temperature, individualized care including dose adjustment is essential.

## Methods

### Database

This study used data from the Japan Medical Data Survey (JAMDAS), a fully anonymized database of electronic health records of general practices in Japan collected by M3, Inc. (Tokyo, Japan) [16]. The JAMDAS database enables real-time extraction of anonymized information entered into the electronic medical records, such as prescription status, drug switching and continuation, laboratory values, clinical evaluation scores, and comorbidities at each clinic [17]. As of November 2023, the database had ~14 million patient records, 11.4% of the nationwide population, and covered ~4700 clinics and 4.5% of general practices nationwide.

### Study design

This is a non-interventional, retrospective cohort study (NCT06604897) using data from the database. A single-arm design was applied for the primary, secondary, and exploratory analysis of the Sac/Val group.

### Institutional review board statement

This study uses data that has been anonymized to prevent the identification of individuals, so it is not classified as personal information under the Act on the Protection of Personal Information. Therefore, the research does not fall under the “Ethical Guidelines for Medical and Biological Research Involving Human Subjects”.

The data originated from the JAMDAS database, which gathers anonymized patient data from various participating clinics. Patients at these clinics were given the chance to opt out before their data was submitted, in accordance with local ethical requirements. The database includes only information that has been anonymized by the clinics themselves, and for which explicit permission for commercial use was granted by each clinic.

The database is managed under strict data governance protocols to ensure the integrity, confidentiality, and ethical use of the information. The investigators received only the aggregated analysis results from M3, Inc., and no identifiable personal data is accessible to researchers. Therefore, the study was deemed exempt from ethics committee or institutional review board approval and the requirement for informed consent. This study complied with all applicable laws regarding subject privacy.

### Participants

The study includes male and female participants ≥18 years of age diagnosed with essential hypertension, BP value on the index date ≥140/90 mmHg, and who were prescribed 100 mg or 200 mg once daily Sac/Val on the index date. Additionally, patients were eligible if their medical record can be extracted 6 months prior to the index date (baseline data), BP data on the index date, and 8 to 12 weeks after the index date can be extracted. Patients with prescription of concomitant ACEi on, after or 2 days before index date were excluded from the study, as well as those diagnosed with angioedema, diabetes and under treatment with Aliskiren fumarate on the index date. Women with a record of pregnancy-related diagnoses, drugs, or medical procedures before or after the index date, or a record of delivery or abortion-related diagnoses, drugs, or medical procedures after the index date were also excluded from the study. Summary of inclusion and exclusion criteria is described in Supplementary Table 1.

### Study period

The data collection period for the Sac/Val group was between March 1, 2021, and October 1, 2022. The period for baseline data were up to 6 months before the index date, corresponding to the date of initial prescription of treatment. The evaluation period was 8 weeks from the index date for primary and secondary analyses and up to 52 weeks from the index date for some of the exploratory analyses.

### Primary objective

The primary objective was to investigate the effectiveness of Sac/Val in lowering BP to achieve individual target levels defined by the JSH2019 [6] at 8 weeks. Target BP thresholds were applied according to patient characteristics as specified in the guideline (e.g., <140/90 mm Hg for older adults). The primary endpoint was determined by the proportion of patients who achieved their target BP within 8 weeks of initial administration of Sac/Val. BP measurements were collected from visits occurring between 8 to 12 weeks, with the data nearest to 8 weeks being used.

### Secondary objectives

Secondary objectives included characterizing the baseline profiles of patients treated with Sac/Val and assessing the relative contribution of these baseline characteristics to achieving BP targets. Additionally, the safety profile of Sac/Val was evaluated by Week 12, focusing on persistence/withdrawal and the incidence of the top three major adverse events listed in the package insert: hypotension, renal function, dehydration, diuresis, and edema or angioedema. Patients discontinued Sac/Val or switched to another medication following those events were also counted as having discontinued the drug.

### Exploratory objectives

Exploratory objectives included the time course of a BP change from baseline to weeks 2, 4, and 8, and every 4 weeks thereafter, as long as the patients are under treatment with Sac/Val and can be followed up (up to 52 weeks); the prescription patterns of Sac/Val stratified by systolic BP (SBP) or diastolic BP (DBP) reduction of ≥10 mmHg from the index date to 8 weeks; and the change in laboratory values from baseline after 8 weeks of drug administration in patients treated with Sac/Val (up to 52 weeks).

### Statistical methods

Demographic and other baseline data including disease characteristics were summarized using descriptive statistics by treatment groups. Definitions of extracting variables are described in Supplementary Table 2. Text mining of free-text fields was performed to allow data extraction including minor symptoms or signs related to hypotension, dehydration, polyuria, and angioedema.

The primary and secondary endpoints indicating proportion analysis were calculated using the proportion of patients and a two-sided 95% confidence interval (CI). Data from patients with a proportion of days covered (PDC) ≥80% were included for the analysis of effectiveness. Statistical testing was performed using paired t-test, two-sample t-test, or χ^2^ test, as appropriate, at a two-sided significance level of 0.05. A paired t-test was used to analyze the mean change in BP and other laboratory values between the index date and each observation. BP or other laboratory values with multiple time points are exploratory and were not adjusted for multiplicity on statistical testing. Pearson’s χ^2^ test or Fisher’s exact test was used to compare the proportion of patients who achieved target BP and mean value of categorical data of demographics.

Multivariate logistic regression analysis was conducted following univariate logistic regression analysis to evaluate the association between each candidate variable and the outcome. The variables included in the univariate analysis were: age (<75/≥75 years), sex (male/female), body mass index (BMI: <18.5/≥18.5, <25.0/≥25.0), cerebrovascular diseases (yes/no), heart diseases (yes/no), diabetes mellitus (yes/no), dyslipidemia (yes/no), renal diseases (yes/no), chronic kidney disease (CKD: yes/no), CKD with proteinuria (yes/no), hypertension grade at the index date (grade I/II/III), and the number of antihypertensive drug classes prescribed prior to sacubitril/valsartan initiation (0/1/2/3–4/≥5). To avoid multicollinearity and enhance the interpretability of the model, BMI, renal diseases, CKD with proteinuria, and the number of antihypertensive drug classes prior to Sac/Val initiation were excluded from the multivariate analysis. These variables were considered to have potential overlap or high correlation with other included clinical factors, which could compromise model stability and obscure the independent effects of each variable. For confounders, we selected those with low *p-*values or high clinical significance. Missing data values were not imputed. If the “day” was unknown, data were handled as 15 days, but if the “year” or “month” data remained unknown, it was taken as a missing value. An SBP ≥300 mmHg or DBP <20 mmHg was considered an absent value. SAS version 9.4 (SAS Institute Inc.) was used for statistical analysis.

## Results

**Fig. 1:**
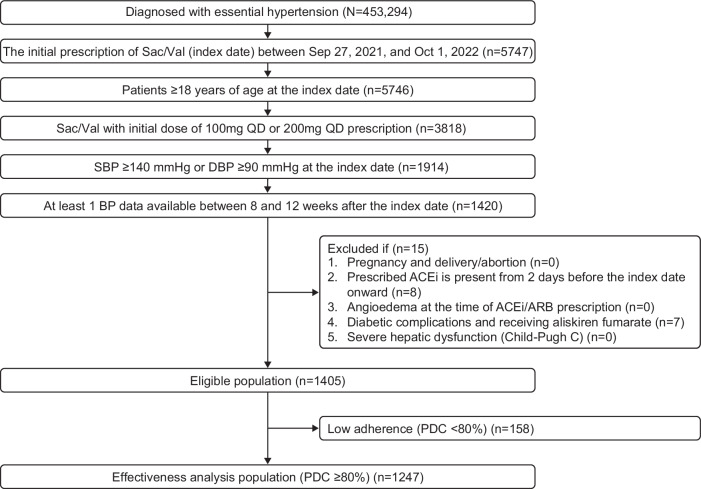
Patients disposition. Effectiveness analysis population was used in the primary analysis and included patients that met the eligibility criteria and had PDC ≥80%. ACEi angiotensin-converting enzyme inhibitor, ARB angiotensin II receptor blockers, DBP diastolic blood pressure, n number of patients, PDC proportion of days covered, QD once a day, Sac/Val sacubitril/valsartan, SBP systolic blood pressure

Of the 1405 eligible patients, 1247 with PDC ≥80% were included in the effectiveness analysis (Fig. 1). The mean age was 71 years, with an almost equal distribution of males (50.4%) and females (49.6%) (Table 1). The mean BMI was calculated for approximately 30% (n = 366) of patients and was 25.6 kg/m^2^, with most patients (n = 186) in the BMI ≥25 group. Average SBP was 158.8 ± 16.2 mmHg and DBP 85.3 ± 14.7 mmHg, with 52% of the patients classified as Grade I hypertension (Table 1). The most common comorbidities were dyslipidemia, followed by heart disease and renal disease (Table 1). A total of 31.1% of patients received two antihypertensive medicines before Sac/Val initiation, whereas 30.6% of patients received 3 to 4 different medicines (Table 1). Most of the patients (55.8%) received CCB, followed by beta-blockers (15.5%) and diuretics (14.3%) at the index date (Table 1). These proportions indicate the frequency with which each drug class was included, either as monotherapy or as part of combination therapy. Of the patients who were prescribed CCB, 55.0% were receiving it in dual therapy with Sac/Val (data not shown).

**Table 1:** Baseline demographic and disease characteristics

	Effectiveness analysis population (N = 1247)	Eligible population (N = 1405)
**Age (years), mean ± SD**	71.5 ± 14.3	71.2 ± 14.4
**Age group (years), n(%)**		
<65	370 (29.7)	432 (30.7)
≥65, <75	259 (20.8)	292 (20.8)
≥75	618 (49.6)	681 (48.5)
**Sex, n(%)**		
Male	629 (50.4)	708 (50.4)
Female	618 (49.6)	697 (49.6)
**BMI (kg/m**^**2**^**), mean ± SD**	25.6 ± 4.6, (n = 366)	25.46 ± 4.64, (n = 405)
**BMI group (kg/m**^**2**^**), n (%)**		
<18	13 (1.0)	17 (1.2)
≥18, <25	167 (13.4)	186 (13.2)
≥25	186 (14.9)	202 (14.4)
**SBP (mmHg), mean ± SD at index date**	158.8 ± 16.2	158.8 ± 16.2
**DBP (mmHg), mean ± SD at index date**	85.3 ± 14.7	85.7 ± 14.7
**Hypertension class at the index date, n (%)**		
Grade I (140–159/90–99 [mmHg])	649 (52.0)	728 (51.8)
Grade II (160–179/100–109 [mmHg])	429 (34.4)	486 (34.6)
Grade III (≥180/110 [mmHg])	169 (13.6)	191 (13.6)
**Comorbidities, n (%)**		
Heart diseases	605 (48.5)	666 (47.4)
Cerebrovascular diseases	264 (21.2)	288 (20.5)
Diabetes mellitus	240 (19.2)	258 (18.4)
Dyslipidemia	750 (60.1)	836 (59.5)
Renal diseases	273 (21.9)	302 (21.5)
CKD	210 (16.8)	236 (16.8)
CKD+ proteinuria	10 (0.8)	11 (0.8)
**Number of antihypertensive drug classes before Sac/Val initiation, n (%)**		
0	200 (16.0)	228 (16.2)
1	256 (20.5)	301 (21.4)
2	388 (31.1)	435 (31.0)
3–4	381 (30.6)	416 (29.6)
≥5	22 (1.8)	25 (1.8)
**Concomitant use of antihypertensive medicines with Sac/Val, n (%)**		
CCB	696 (55.8)	775 (55.2)
Beta-blocker	193 (15.5)	211 (15.0)
Diuretics	178 (14.3)	195 (13.9)
MRA	120 (9.6)	132 (9.4)
Alpha-beta-blocker	89 (7.1)	95 (6.8)
Alpha-blocker	49 (3.9)	56 (4.0)
ARB	46 (3.7)	54 (3.8)
None	372 (29.8)	431 (30.7)

*ARB* angiotensin II receptor blocker, *BMI* body mass index, *BP* blood pressure, *CCB* calcium channel blocker, *CKD* chronic kidney disease, *DBP* diastolic blood pressure, *MRA* mineralocorticoid antagonist,

*Sac/Val* sacubitril/valsartan, *SBP* systolic blood pressure, *SD* standard deviation

### Primary endpoint

**Fig. 2:**
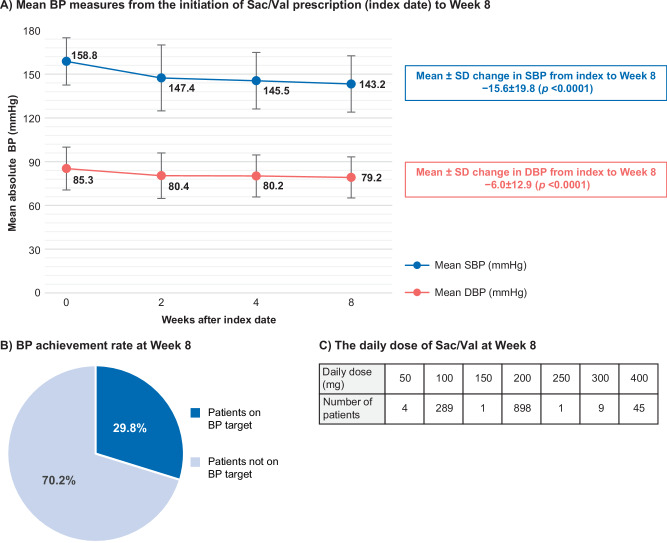
Blood pressure reduction over time and target BP achievement rate. **A** Graph showing mean BP measures from the initiation of Sac/Val prescription (index date) to Week 8; **B** Pie chart showing the achievement rate of target BP; **C** Table summarizing daily dose of Sac/Val at Week 8 from index date. DBP diastolic blood pressure, BP blood pressure, Sac/Val sacubitril/valsartan, SBP systolic blood pressure, SD standard deviation

Sac/Val treatment reduced SBP, DBP, and pulse pressure throughout the study period (Fig. 2 and Supplementary Fig. 1). A total of 29.8% (n = 371) patients treated with Sac/Val achieved the individual estimated JSH2019-recommended antihypertensive goal (Fig. 2). Treatment with Sac/Val caused a significant mean reduction of −15.6 mmHg and −6.1 mmHg for SBP and DBP, respectively (*p* < 0.0001; Fig. 2A). The daily dose of Sac/Val at the endpoints is presented in Fig. 2C.

### Secondary endpoints

Multivariate logistic regression analysis revealed that patients classified as Grade II and Grade III hypertension at index date had reduced likelihood (odds ratio [OR]: 0.45 [95% CI: 0.33–0.60] and OR: 0.24 [95% CI: 0.15–0.39]) of reaching the estimated antihypertensive goals compared to those classified as Grade I hypertension (Table 2). Patients aged ≥75 years (OR: 3.65 [95% CI: 2.71–4.93], *p* < 0.0001) and patients with cerebrovascular disease (OR: 1.71 [1.25–2.33], *p* = 0.0007) were more likely to meet the estimated antihypertensive goals compared to younger patients and those without this condition (Table 2).

**Table 2:** Results of logistic regression analysis of variables on target BP attainment (effectiveness analysis population)

Parameter		n	Achievement n (%)	Univariate analysis		Multivariate analysis	
				Odds ratio (95% CI)	p-value	Odds ratio (95% CI)	p-value
**Age (years)**							
<75 (ref)		629	106 (16.9)	3.70 (2.85–4.82)	<0.0001	3.65 (2.71–4.93)	<0.0001
≥75		618	265 (42.9)				
**Sex**							
Male (ref)		629	175 (27.8)	1.20 (0.94–1.54)	0.1329	0.83 (0.63–1.09)	0.1874
Female		618	196 (31.7)				
**BMI (kg/m**^**2**^**)**							
<18.5 (ref)		13	4 (30.8)	-	-	-	-
≥18.5, <25.0		167	64 (38.3)	1.40 (0.41–4.73)	0.5898	-	-
≥25.0		186	43 (23.1)	0.68 (0.20–2.31)	0.5325	-	-
**Comorbidities**^a^							
Cerebrovascular diseases	No	983	260 (26.4)	2.02 (1.52–2.68)	<0.0001	1.71 (1.25–2.33)	0.0007
	Yes	264	111 (42.0)				
Heart diseases	No	642	152 (23.7)	1.83 (1.43–2.34)	<0.0001	1.24 (0.94–1.64)	0.1287
	Yes	605	219 (36.2)				
Diabetes mellitus	No	1007	307 (30.5)	0.83 (0.60–1.14)	0.2452	0.83 (0.58–1.17)	0.2828
	Yes	240	64 (26.7)				
Dyslipidemia	No	497	140 (28.2)	1.13 (0.88–1.46)	0.3199	0.87 (0.68–1.15)	0.3193
	Yes	750	231 (30.8)				
Renal diseases	No	974	277 (28.4)	1.32 (0.99–1.76)	0.0560	-	-
	Yes	273	94 (34.4)				
CKD	No	1037	297 (28.6)	1.36 (0.99–1.85)	0.0571	0.96 (0.68–1.36)	0.8247
	Yes	210	74 (35.2)				
CKD+ proteinuria	No	1237	367 (29.7)	1.58 (0.44–5.63)	0.4802	-	-
	Yes	10	4 (40.0)				
**Hypertension class at the index date**							
Grade I (ref)		649	250 (38.5)	-	-	-	-
Grade II		429	98 (22.8)	0.47 (0.36–0.62)	<0.0001	0.45 (0.33–0.60)	<0.0001
Grade III		169	23 (13.6)	0.25 (0.16–0.40)	<0.0001	0.24 (0.15–0.39)	<0.0001
**Number of antihypertensive drug classes prior to Sac/Val initiation**							
0 (ref)		200	64 (32.0)			-	-
1		256	76 (29.7)	0.90 (0.60–1.34)	0.5953	-	-
2		388	111 (28.6)	0.85 (0.59–1.23)	0.3943	-	-
3–4		381	113 (29.7)	0.90 (0.62–1.30)	0.5603	-	-
≥5		22	7 (31.8)	0.99 (0.39–2.55)	0.9862	-	-

*BMI* body-mass index, *BP* blood pressure, *CI* confidence interval, *CKD* chronic kidney disease, *Sac/Val* sacubitril/valsartan

^a^ref. is the number of patients without the respective comorbidity

Analysis of prescription patterns (Table 3) showed that among patients with BP reduction of ≥10 mmHg, most were prescribed CCB+Sac/Val combination on the index date (29.9% with SBP reduction ≥10 mmHg and 26.9% with DBP reduction ≥10 mmHg). Of those, a majority were on CCB + ARB combination therapy and switched to CCB+Sac/Val (51.5% with SBP reduction ≥10 mmHg and 48.4% with DBP reduction ≥10 mmHg), followed by patients who were on CCB monotherapy (16.0% with SBP reduction ≥10 mmHg and 16.1% with DBP reduction ≥10 mmHg).

**Table 3:** Prescription patterns for patients with BP reductions ≥10 mmHg (effectiveness analysis population)

Prescription pattern before index date	Prescription pattern at index date	Patients with SBP reduction ≧10 mmHg, n (%) (N = 897)	Proportion in CCB+Sac/Val (%)	Patients with DBP reduction ≧10 mmHg, n (%) (N = 598)	Proportion in CCB+Sac/Val (%)
Any	CCB + Sac/Val	268 (29.9)	100	161 (26.9)	100.0
CCB + ARB	CCB + Sac/Val	138 (15.4)	51.5	78 (13.0)	48.4
CCB	CCB + Sac/Val	43 (4.8)	16.0	26 (4.3)	16.1
Others	CCB + Sac/Val	87 (9.7)	32.5	57 (9.5)	35.4

*ARB* angiotensin receptor blocker, *BP* blood pressure, *CCB* calcium channel blocker, *DBP* diastolic blood pressure, *Sac/Val* sacubitril/valsartan, *SBP* systolic blood pressure

### Safety endpoints

The most frequently observed adverse events (AEs) were signs of hypotension and signs of diuresis-related events (dehydration and polyuria), occurring at rates of 8.4% and 7.8% (3.6% and 4.2%), respectively, and the rates of corresponding AEs with discontinuation were 1.0% and 0.8% (0.3% and 0.5%), respectively (Table 4A). Many of these patients were taking diuretics at the time of the events (35.1%, 40.8%, and 49.4%, respectively) (Table 4B), and the proportion of patients with a history of renal disease in those patients (25.4%, 36.0%, and 22.0%, respectively) was higher than in total eligible patients (21.5%) (Supplementary Table 3, Table 1). Patients who were suspected of having symptoms of hypotension or dehydration were more likely to have a history of cerebrovascular disease (31.5% and 26.9%, respectively) than total eligible patients (20.5%) (Supplementary Table 3 and Table 1). During summer, patients had a higher probability of showing signs of hypotension and dehydration, with a total of 60 (4.3%) and 37 (2.6%) events, respectively (Table 4B).

**Table 4:** Summary of key safety events. (A) Safety events at 8 weeks after the index date (eligible population). (B) Safety events per season and concomitant diuretic use (eligible population)

A								
	Number of events	Number of patients with event	Sac/Val dose at events					Incidence rate (95% CI)
			50 mg	100 mg	200 mg	250 mg	400 mg	
**Hypotension**	185	118	1	47	134	0	3	8.4 (7.0–10.0)
*discontinued patients*	24	14	0	10	14	0	0	1.0 (0.5–1.7)
**Dehydration**	76	50	0	24	49	1	2	3.6 (2.7–4.7)
*discontinued patients*	5	4	0	2	3	0	0	0.3 (0.1–0.7)
**Polyuria**	87	59	1	26	60	0	0	4.2 (3.2–5.4)
*discontinued patients*	14	7	0	1	13	0	0	0.5 (0.2–1.0)
**Angioedema**	0	0	0	0	0	0	0	0.0 (0.0–0.3)
*discontinued patients*	0	0	0	0	0	0	0	0.0 (0.0–0.3)
**Renal outcomes**	47	47	0	17	29	0	1	3.3 (2.5–4.4)
*discontinued patients*	3	3	0	1	2	0	0	0.2 (0.0–0.6)
**New onset of renal disease**	34	34	0	10	23	0	1	2.4 (1.7–3.4)
*discontinued patients*	3	3	0	1	2	0	0	0.2 (0.0–0.6)
**New onset of CKD**	38	38	0	12	25	0	1	2.7 (1.9–3.7)
*discontinued patients*	3	3	0	1	2	0	0	0.2 (0.0–0.6)
**≥40% eGFR reduction from the index date**	7	7	0	2	5	0	0	0.5 (0.2–1.0)
*discontinued patients*	1	1	0	0	1	0	0	0.1 (0.0–0.4)
**New onset of UACR** ≥**30 mg/gCr or UPCR** ≥**0.15 g/gCr**	6	6	0	3	3	0	0	0.4 (0.2–0.9)
*discontinued patients*	0	0	0	0	0	0	0	0.0 (0.0–0.3)

B								
Safety Event	Season	Events n (%)	Concomitant diuretics use per events n (%)	Sac/Val daily dose at events, n				
				50 mg	100 mg	200 mg	250 mg	400 mg
**Hypotension**	All year	185 (13.2)	65 (35.1)	1	47	134	0	3
	Spring	47 (3.3)	13 (27.7)	1	7	39	0	0
	Summer	60 (4.3)	24 (40.0)	0	21	39	0	0
	Autumn	47 (3.3)	17 (36.2)	0	13	33	0	1
	Winter	31 (2.2)	11 (35.5)	0	6	23	0	2
**Dehydration**	All year	76 (5.4)	31 (40.8)	0	24	49	1	2
	Spring	13 (9.3)	6 (46.2)	0	3	9	0	1
	Summer	37 (2.6)	14 (37.8)	0	13	24	0	0
	Autumn	22 (1.6)	10 (45.5)	0	6	14	1	1
	Winter	4 (0.3)	1 (25.0)	0	2	2	0	0
**Polyuria**	All year	87 (6.2)	43 (49.4)	1	26	60	0	0
	Spring	26 (1.9)	16 (61.5)	1	8	17	0	0
	Summer	22 (1.6)	13 (59.1)	0	5	17	0	0
	Autumn	20 (1.4)	8 (40.0)	0	5	15	0	0
	Winter	19 (1.4)	6 (31.6)	0	8	11	0	0

*CI* confidence interval, *CKD* chronic kidney disease, *UACR* urine albumin-creatinine ratio, *UPCR* urine protein-creatinine ratio, *Sac/Val* sacubitril/valsartan

Spring: March to May, Summer: June to August, Autumn: September to November, Winter: December to February. Sac/Val, sacubitril/valsartan

No significant change was observed in laboratory values from index date to Week 8 for eGFR (−1.0 ± 7.6, *p* = 0.1385), potassium (−0.08 ± 0.55, *p* = 0.1226), and HbA1c (−0.16 ± 0.68, *p* = 0.0635). This trend continued until the end of the study (Week 52) (Supplementary Fig. 2).

## Discussion

Uncontrolled BP is a significant risk factor for cardiovascular disease [18], and raising awareness, intensifying antihypertensive treatment, and improving adherence can help achieve recommended BP goals [2]. In Japan, approximately 73% of individuals with hypertension do not receive adequate management. Among them, 33% are unaware of their condition, 11% are aware but not receiving any treatment, and 29% are under-treated [2]. A recent retrospective observational study showed that switching to Sac/Val or using Sac/Val as an add-on to other antihypertensive drugs is beneficial for patients with poorly controlled or untreated hypertension [19].

The present study verifies the real-world effectiveness of Sac/Val and identifies patient characteristics that predict better achievement of BP targets with Sac/Val, as well as treatment patterns that may enhance the efficiency of lowering BP. Nearly one-third of the patients reached their JSH2019-recommended BP goals, with reductions of 15.6 mmHg in SBP and 6.1 mmHg in DBP. This aligns with a recent real-world study in patients who were undertreated or had poorly controlled hypertension, which showed that switching to or adding Sac/Val lowered SBP by 11.7 mmHg and DBP by 5.7 mmHg [19]. Patients with Grade I hypertension were more likely to meet the recommended antihypertensive goals than patients with Grade II and Grade III hypertension. In addition, among elderly patients aged ≥75 years and those with cerebrovascular disease, the achievement rate of the recommended BP targets was higher than younger patients aged <75 years and those without such comorbidity. We considered that one of the main reasons why patients aged ≥75 years emerged as a key factor is that the BP target specified in the JSH2019, which set a 10 mmHg higher threshold for patients in this age group and appears to have a notable impact on the clinical outcomes [5, 6]. In a randomized controlled trial (RCT) of Sac/Val, about two-fifths of patients achieved <140/90 mmHg and approximately one-half of those achieved <130/80 mmHg [8].

The association with cerebrovascular comorbidity may reflect the preferential use of intensive antihypertensive treatment in these patients, as supported by meta-analyses of clinical trials showing superior efficacy in stroke prevention [20, 21].

Many real-world studies involving patients with poorly controlled BP have proven the greater antihypertensive effects of Sac/Val compared to ARBs, leading to frequent switches from ARBs to Sac/Val [8, 10–12, 19]. Furthermore, Sac/Val is also used for transitioning from other classes of antihypertensive drugs, including combination drugs, and can be used as an additional drug in the treatment of hypertension [19]. In this analysis, the most frequent prescription pattern for patients who experienced a reduction in both SBP and DBP by more than 10 mmHg was a combination of Sac/Val with CCB, and these patients mostly switched from a combination of ARB with CCB. These observations align with a recent real-world study that demonstrated similar treatment trends, indicating that incorporating Sac/Val in conjunction with or as a replacement for various antihypertensive medications effectively further reduced BP in patients with poorly managed or untreated hypertension [19]. Given that a reduction of 10 mmHg was set as the cutoff value in our analysis, we did not assess the extent of BP reduction among patients who experienced decreases exceeding this threshold. Therefore, the present findings should not be interpreted as evidence that switching from a CCB + ARB regimen to CCB+Sac/Val yields the greatest antihypertensive effect among those with reductions ≥10 mmHg. Although previous RCTs have suggested that the addition of Sac/Val to CCB monotherapy may result in a greater BP reduction than substituting ARB with Sac/Val in a CCB + ARB combination [8, 10, 11, 22, 23], our results imply that, in real-world clinical practice, Sac/Val is more commonly introduced by switching from a CCB + ARB regimen rather than by adding it to CCB monotherapy.

Seasonal variations in BP related to ambient temperature have been observed in the general population, with a decrease in BP typically occurring during the summer months [24–27]. Additionally, it was reported that orthostatic hypotension was more frequent in summer compared with winter in hypertensive patients under antihypertensive therapy [28]. We observed the same trend in this study and the recorded low discontinuation rate of 1% after the observation suggests that in real-world clinical practice, physicians were able to identify the signs of hypotension (dizziness, vertigo and fainting) timely and manage them adequately.

In addition to orthostatic hypotension, dehydration occurs more frequently in patients under antihypertensive treatment during summer [29]. Similarly, in this study, signs of dehydration such as thirst and dry mouth were also recorded more frequently during this season. The proportion of concomitant diuretic use in patients who experienced signs of dehydration was higher than in the eligible population in all seasons. Sac/Val has neprilysin enzyme inhibition effects and increases natural natriuretic peptides (NPs) [30, 31], which have natriuretic and diuretic effects [32, 33]. In a RCT of Sac/Val for patients with salt-sensitive hypertension, treatment with Sac/Val resulted in only a short-term initial effect of natriuresis and diuresis within 24 h [34]. This effect was not observed after 28 days of daily Sac/Val administration, which may be due to NP-derived vasodilatory effects and initial natriuresis that could have restored sodium and water balance and thus prevented sustained natriuresis and diuresis [34]. This is consistent with previous studies investigating the sodium sensitivity in patients with essential hypertension [35]. Given these considerations, if diuretics are used in combination with Sac/Val and dehydration is a concern, it may be helpful to first reduce the dose of the diuretic that causes forced diuresis. Although the number of discontinuations in this study was small, it is advisable that physicians continuously monitor for dehydration, as it can cause hypotension and organ injuries.

It may be helpful to seasonally review drug dosages, including concomitant medications, especially diuretics, to avoid hypotension and to also monitor fluid intake to prevent dehydration, particularly in summer.

### Study limitations

The study used a clinic-based electronic health record database, so it is not possible to track if patients were transferred to other clinics and some clinical data may have been missing or incorrectly recorded. Due to the retrospective nature of the study, BP may have been measured in different settings, such as waiting rooms, clinic offices, or patient’s home, which could have influenced the results. Additionally, the actual BP goals set for individual patients by physicians are unknown. It was also not possible to determine whether the patient collected their prescription or used their medication as recommended. Selection bias may also exist as the study population was derived from a database covering a limited proportion of clinics in Japan, and further narrowed by specific inclusion/exclusion criteria such as medication adherence. Additionally, to ensure that the study population consisted of patients with hypertension, we defined eligibility using both ICD-10 codes and elevated BP values (≥140/90 mmHg). As a result, patients whose BP had already been controlled below this threshold through prior antihypertensive treatment may have been excluded. These factors may limit the generalizability of the findings to the broader hypertensive population. Safety profiles were extracted using International Classification of Diseases-10 (ICD-10) codes and text mining, which is not validated and may result in over- or under-reporting. Dialysis data were not available at the time of data extraction for the study.

### Perspective of Asia

Hypertension risk increases with age [36], and Asia’s rapidly aging population suggests a growing predominance of elderly hypertensive patients. Because older individuals exhibit greater BP changes in response to ambient temperature, individualized care including dose adjustment is essential [37]. Future studies are warranted to establish evidence-based strategies for optimizing titration of combination therapies including Sac/Val to ensure safety and efficacy in this population.

## Conclusions

In real-world clinical settings in Japan, the treatment incorporating Sac/Val has been found to be effective in achieving guideline-recommended target BP and safe in patients with hypertension. Patients aged ≥75 years, those with cerebrovascular disease, and those classified as Grade I hypertension were more likely to benefit from the treatment. The combination therapy of CCB and Sac/Val is the most frequently observed among patients who showed a marked response (BP reduction in ≥10 mmHg) in the real world. Additionally, this analysis highlights that continued revision of drug dosages, including concomitant medications, and monitoring signs of hypotension and dehydration carefully, especially in summer, will benefit the patients. Following the recent update of the JSH guidelines, which set a uniform target of <130/80 mmHg for all patients with tolerability, including those aged 75 years and older, the next challenge is to evaluate whether Sac/Val can further contribute to the safe implementation of stricter blood pressure control.

## Supplementary information

Supplementary information

## Data Availability

Novartis Pharma K.K. is committed to sharing access to analysis results and supporting clinical documents from eligible studies with qualified external researchers. For this study, data were anonymized and analyzed by M3, Inc. and the research group only received analysis results. Due to a contract between Novartis Pharma K.K. and M3, Inc., the data set used in this study cannot be shared, but the original database is available from M3, Inc. upon reasonable request. All data provided are anonymized to respect the privacy of patients who have participated in the trial in line with applicable laws and regulations. This trial data availability is according to the criteria and process described on www.clinicalstudydatarequest.com.
