# Supplementary material for: Impact of treatment strategies incorporating sacubitril/valsartan on achievement of guideline-recommended blood pressure targets and representative safety outcomes
Source: Hypertens Res. 2026 Jan 14;49(4):1232–44. doi: 10.1038/s41440-025-02537-w (PMC13050650; doi:10.1038/s41440-025-02537-w)
Supplement: Supplementary file 1 — Supplementary information [file 41440_2025_2537_MOESM1_ESM.docx]

| **Supplementary Table 1**: Participant inclusion and exclusion criteria |
| --- |
| **Inclusion criteria** |
| - Age ≥18 years on the index date* - Diagnosed with essential hypertension; ICD-10 codes: I10 on the index date - Patients for whom data for the 6 months prior to the index date can be extracted as baseline data - Patients for whom BP data can be extracted between 8 and 12 weeks after the index date - Patients whose BP data can be extracted on the index date - Patients whose BP value on the index date was ≥140/90 mmHg - Patients with the prescription of 100 mg or 200 mg once daily by 100-mg or  200-mg tablets of Sac/Val on the index date |
| **Exclusion criteria** |
| - Women with a record of pregnancy-related diagnoses, drugs, or medical procedures before or after the index date, or a record of delivery- or abortion-related diagnoses, drugs, or medical procedures after the index date - Prescription of concomitant ACEi (ATC code: C09A) is present from 2 days before the index date on ward - Patients who are diagnosed with angioedema(ICD-10: D84.1) under prescription of ACEi or ARBs; ATC code: C09C - Patients who are diagnosed with diabetes and under treatment with aliskiren fumarate on index date - Patients with severe impaired liver functions with Child-Pugh classification C |
| *The index date for Sac/Val group will be the date of initial prescription of Sac/Val; the index date for conventional drug-control groups will be the date of initial treatment defined as active-comparators.  ACEi, angiotensin-converting enzyme inhibitors; ATC, anatomical therapeutic chemical; ARB, angiotensin receptor blocker; BP, blood pressure; ICD, international classification of diseases; Sac/Val, sacubitril/valsartan. |

| **Supplementary Table 2:** Definition of extracting variables (ACT and ICD-10 codes) |
| --- |
| **Antihypertensive medications** |
| - CCB [C08C] - ACEi [C09A] - ARB [C09C] - Diuretic [C03A, C03B, C03C] - β-blocker [C07AB] - α-blocker [C02CA] - αβ-blocker [C07AG] - Aldosterone antagonist [C03DA, C03DB] - Renin blocker [09XA] - Single pill combination [C02N, C02L, C07BB, C07BG, C07CB, C07CG, C07D, C03E, C07FB, C07FX01, C08G, C09B, C09D, C09XA52, C09XA53, C09XA54] - Sacubitril/valsartan [D10226] |
| **Cerebrovascular disease** |
| - Cerebrovascular disorder [I60-I69] - Vertebral artery thrombosis* [I650] - Vertebral artery occlusive disease* [I650] - Basilar artery thrombosis* [I651] - Basilar artery occlusion* [I651] - Internal carotid artery thrombosis* [I652] - Internal carotid artery occlusion* [I652] - Middle cerebral artery thrombosis* [I660] - Middle cerebral artery occlusion* [I660] - Anterior cerebral artery thrombosis* [I661] - Anterior cerebral artery occlusion* [I661] - Posterior cerebral artery thrombosis* [I662] - Posterior cerebral artery occlusion* [I662]   ** Defined as occlusion of the main cerebral artery* |
| **Heart disease** |
| - Rheumatic fever with cardiac complications [I01] - Chronic rheumatic heart disease [I05-I09] - Angina pectoris* [I20] - Acute myocardial infarction* [I21] - Recurrent myocardial infarction* [I22] - Other acute ischemic heart disease* [I24] - Chronic ischemic heart disease* [I25] - Other types of heart disease [I30-I52]   **Defined as coronary artery disease* |
| **Diabetes mellitus** |
| - Type 1 insulin-dependent diabetes mellitus (IDDM) [E10] - Type 2 insulin-independent diabetes mellitus (NIDDM) [E11] |
| **Dyslipidemia** |
| - Lipoprotein metabolism disorders and other lipemia [E78] |
| **Renal disease** |
| - Glomerular diseases [N00-N08] - Renal tubulointerstitial disease [N10-N16] - Acute renal failure [N17] - Chronic kidney disease (CKD) [N18],   - eGFR <60, UACR ≥30 mg/gCr (with spot urine)*, or UPCR ≥0.15 g/gCr (with spot urine)* - Renal failure of unknown details [N19] - Hypertensive renal disease [I12] - Hypertensive cardio-renal disease [I13]   **Defined as proteinuria* |
| **Renal outcomes** |
| - If any of the following apply after Day 0, included in renal outcome counts:   - Renal diseases   - CKD   - ∆eGFR (percent decrease from baseline) ≥40% |
| **Hypotension-related events** |
| - Orthostatic hypotension [I951] - Postural hypotension [I951] - Secondary orthostatic hypotension [I951] - Hypertension due to the drugs [I952] - Episodic hypotension [I959] - Hypotension [I959] |
| **Dehydration-related events** |
| - Dehydration [E86] - Isotonic dehydration [E86] |
| **Diuresis-related events** |
| - Polyuria [R35] - Idiopathic polyuria [R35] - Frequent urination [R35] - Nocturnal polyuria [R35] - Nocturia [R35] |
| **Angioedema-related events** |
| - Angioneurotic edema [T783] - Quincke's edema [T783] |
| ACEi, angiotensin-converting enzyme inhibitor; ATC, anatomical therapeutic chemical; ARB, angiotensin receptor blocker; CCB, calcium channel blocker; CKD, chronic kidney disease; eGFR, estimated glomerular filtration rate; ICD, international classification of diseases; UACR, urine albumin-creatinine ratio; UPCR, urine protein-creatinine ratio. |

| **Supplementary Table 3.** Comorbidities and BP category of patients who experienced safety event per season (eligible population) | | | | | | | | | | | | |
| --- | --- | --- | --- | --- | --- | --- | --- | --- | --- | --- | --- | --- |
| **Safety Event** | **Season** | **Patients with event, n** | **Concomitant diuretic use per patient,  n (%)** | **Comorbidity at baseline, n (%)** | | | | | | **BP category, n (%)** | | |
|  |  |  |  | **CeVD** | **Heart disease** | **Diabetes** | **Dyslipidemia** | **Renal disease** | **CKD** | **Grade  I** | **Grade  II** | **Grade III** |
| **Hypotension** | All year | 118 | 38  (32.2) | 36  (30.5) | 63  (53.4) | 24  (20.3) | 73  (61.9) | 30  (25.4) | 20  (16.9) | 66 (55.9) | 34 (28.8) | 18 (15.3) |
|  | Spring | 29 | 9  (31.0) | 9  (31.0) | 17  (58.6) | 7  (24.1) | 20  (69.0) | 7  (24.1) | 4  (13.8) | 19 (65.5) | 8  (27.6) | 2  (6.9) |
|  | Summer | 38 | 12  (31.6) | 12  (31.6) | 21  (55.3) | 10  (26.3) | 21  (55.3) | 9  (23.7) | 7  (18.4) | 18 (47.4) | 15 (39.5) | 5  (13.2) |
|  | Autumn | 30 | 12  (40.0) | 8  (26.7) | 15  (50.0) | 5  (16.7) | 17  (56.7) | 8  (26.7) | 5  (16.7) | 18 (60.0) | 6  (20.0) | 6  (20.0) |
|  | Winter | 21 | 5  (23.8) | 7  (33.3) | 10  (47.6) | 2  (9.5) | 15  (71.4) | 6  (28.6) | 4  (19.0) | 11 (52.4) | 5  (23.8) | 5 (23.8) |
| **Dehydration** | All year | 50 | 20  (40.0) | 13  (26.0) | 24  (48.0) | 7  (14.0) | 26  (52.0) | 18  (36.0) | 15  (30.0) | 29 (58.0) | 18 (36.0) | 3  (6.0) |
|  | Spring | 10 | 4  (40.0) | 2  (20.0) | 5  (50.0) | 0  (0.0) | 5  (50.0) | 3  (30.0) | 2  (20.0) | 7  (70.0) | 3  (30.0) | 0  (0.0) |
|  | Summer | 26 | 10  (38.5) | 7  (26.9) | 11  (42.3) | 4  (15.4) | 15  (57.7) | 11  (42.3) | 9  (34.6) | 13 (50.0) | 11 (42.3) | 2  (7.7) |
|  | Autumn | 10 | 5  (50.0) | 2  (20.0) | 5  (50.0) | 2  (20.0) | 5  (50.0) | 3  (30.0) | 3  (30.0) | 7 (70.0) | 3  (30.0) | 0  (0.0) |
|  | Winter | 4 | 1  (25.0) | 2  (50.0) | 3  (75.0) | 1  (25.0) | 1  (25.0) | 1  (25.0) | 1  (25.0) | 2 (50.0) | 1  (25.0) | 1  (25.0) |
| **Polyuria** | All year | 59 | 28  (47.5) | 13  (22.0) | 31  (52.5) | 9  (15.3) | 38  (64.4) | 13  (22.0) | 11  (18.6) | 28 (47.5) | 24 (40.7) | 7  (11.9) |
|  | Spring | 18 | 9  (50.0) | 5  (27.8) | 10  (55.6) | 2  (11.1) | 11  (61.1) | 5  (27.8) | 5  (27.8) | 8  (44.4) | 7  (38.9) | 3  (16.7) |
|  | Summer | 16 | 8  (50.0) | 2  (12.5) | 9  (56.3) | 3  (18.8) | 9  (56.3) | 3  (18.8) | 2  (12.5) | 6  (37.5) | 8  (50.0) | 2  (12.5) |
|  | Autumn | 15 | 6  (40.0) | 4  (26.7) | 8  (53.3) | 3  (20.0) | 10  (66.7) | 4  (26.7) | 3  (20.0) | 7  (46.7) | 7  (46.7) | 1  (6.7) |
|  | Winter | 10 | 5  (50.0) | 2  (20.0) | 4  (40.0) | 1  (10.0) | 8  (80.0) | 1  (10.0) | 1  (10.0) | 7  (70.0) | 2  (20.0) | 1  (10.0) |

Spring: March to May, Summer: June to August, Autum: September to November, Winter: December to February. BP, blood pressure; CeVD, cerebrovascular disease; CKD, chronic kidney disease.

**
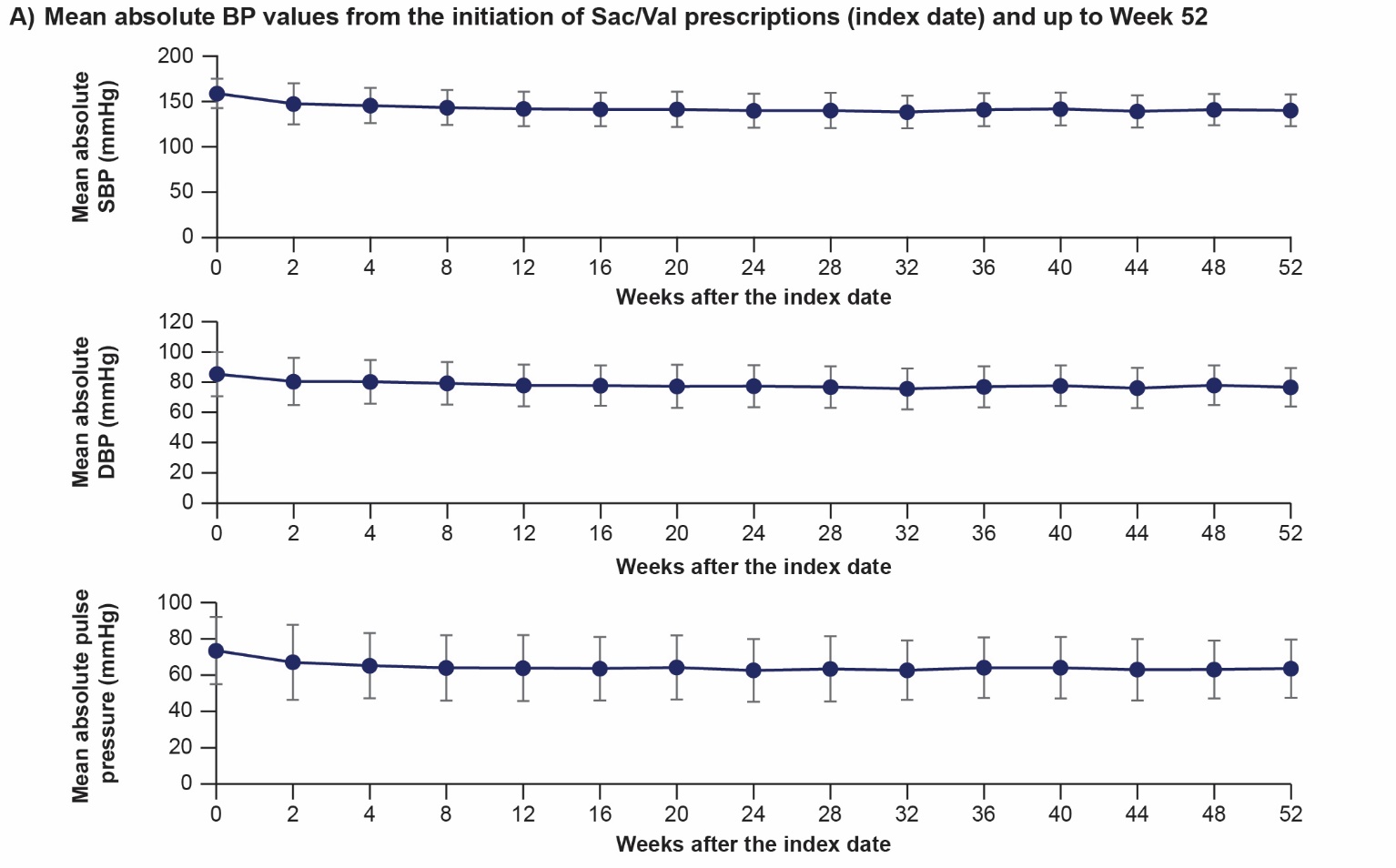
**

**
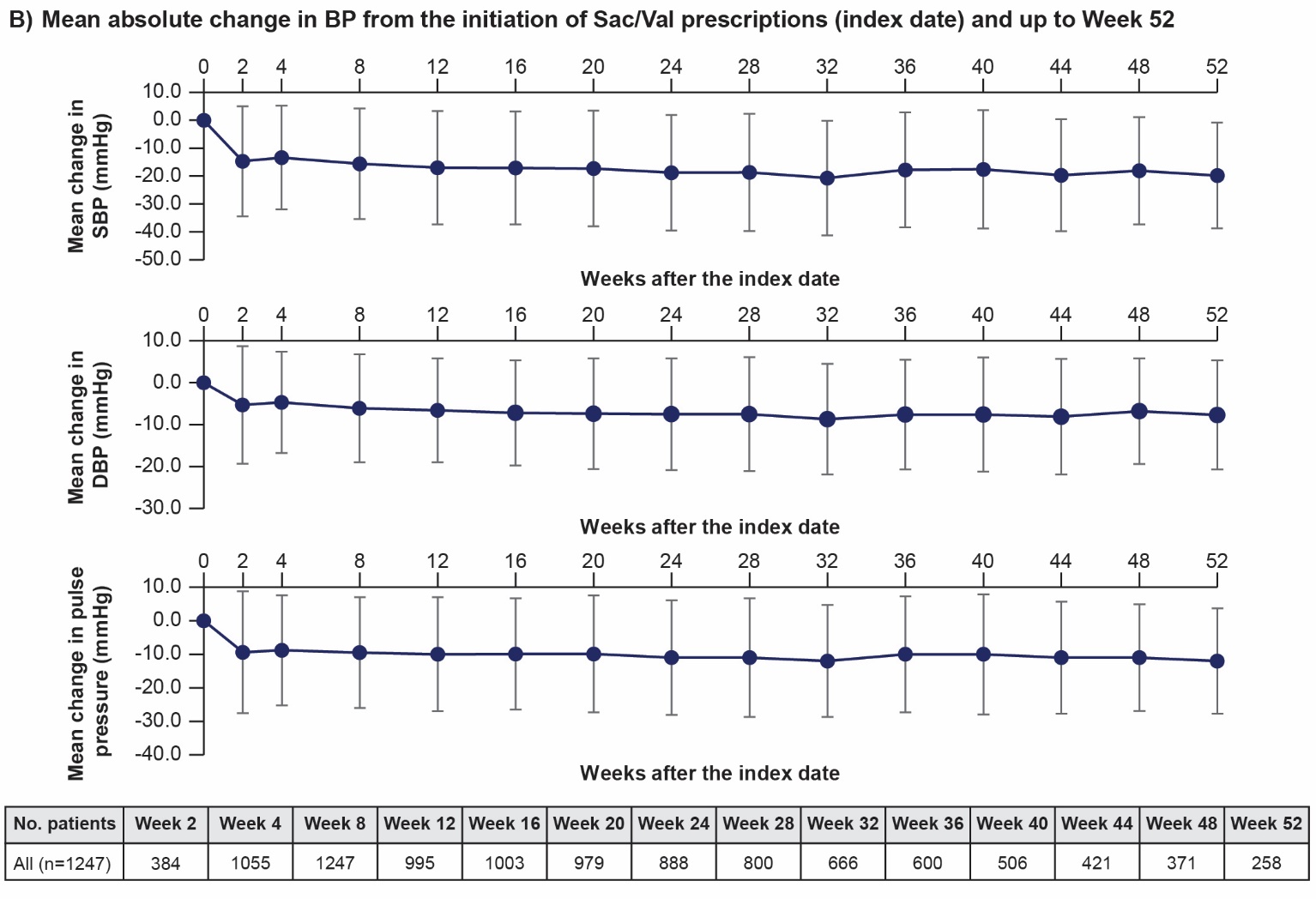
**

**Supplementary Figure 1.** Change in BP over time in effectiveness analysis population from the initiation of Sac/Val prescriptions (index date) and up to Week 52. A) Mean absolute BP values from index date and up to Week 52; B) Mean absolute change in BP from index date and up to Week 52. Abbreviations: BP, blood pressure, DBP, diastolic blood pressure; Sac/Val, sacubitril/valsartan; SBP, systolic blood pressure.


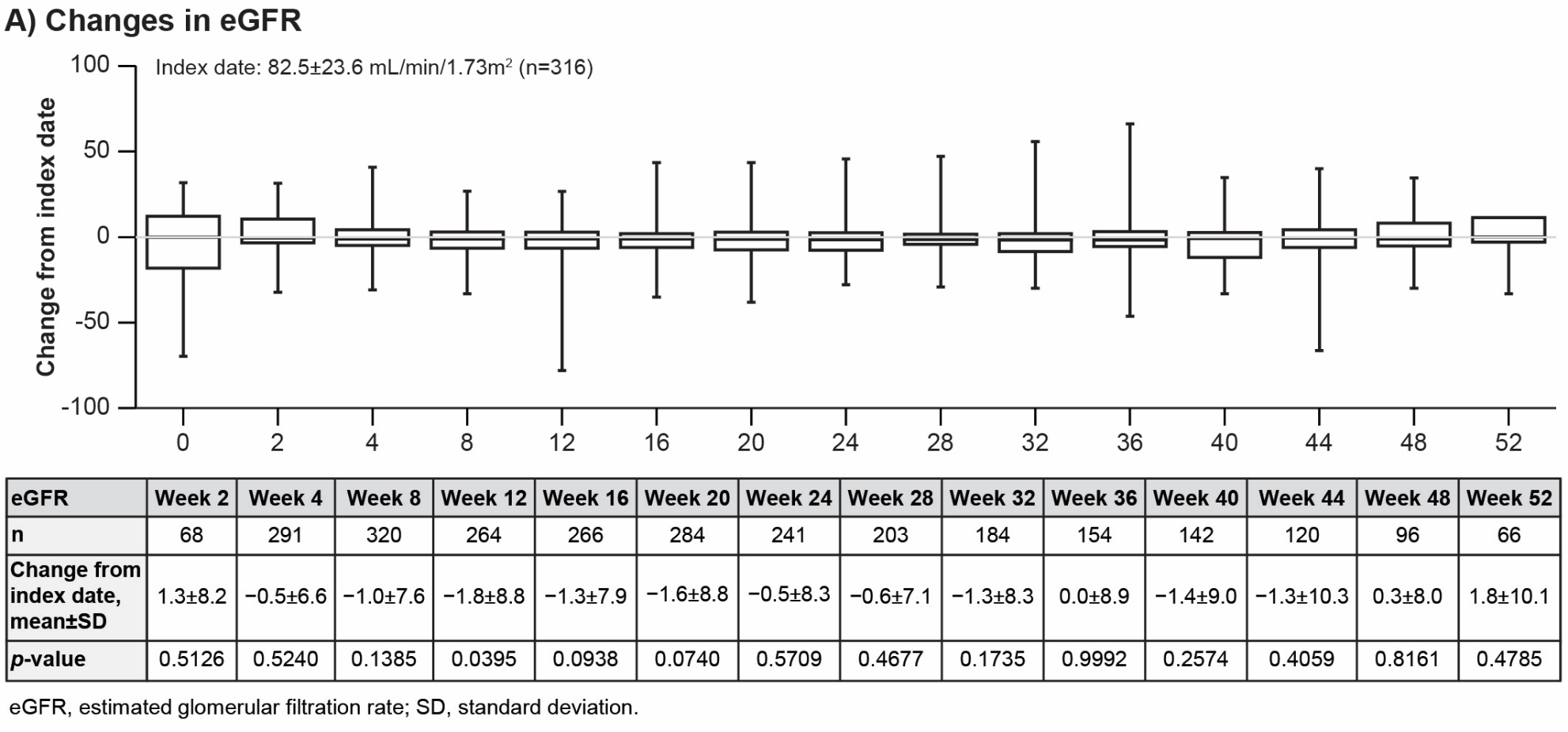


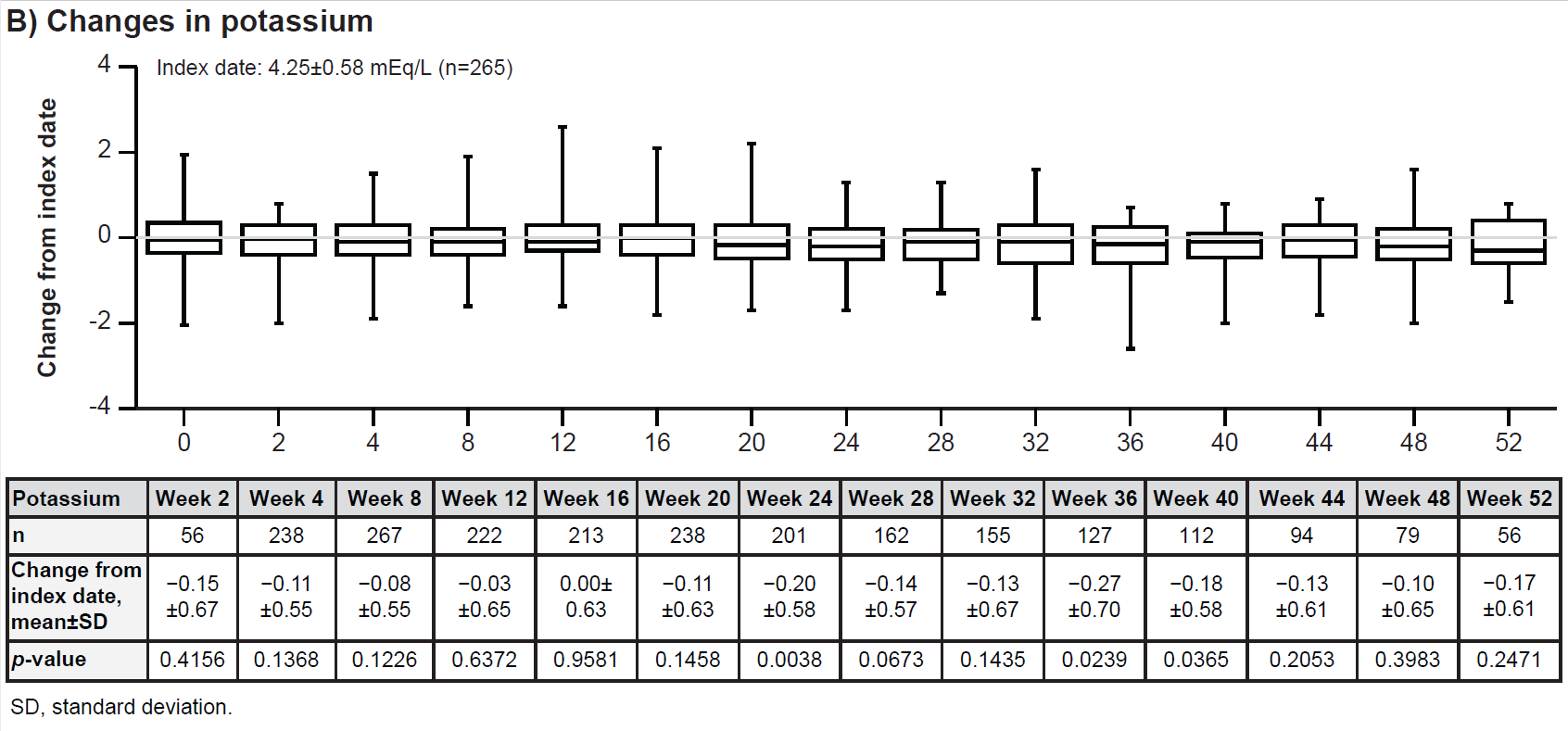


**
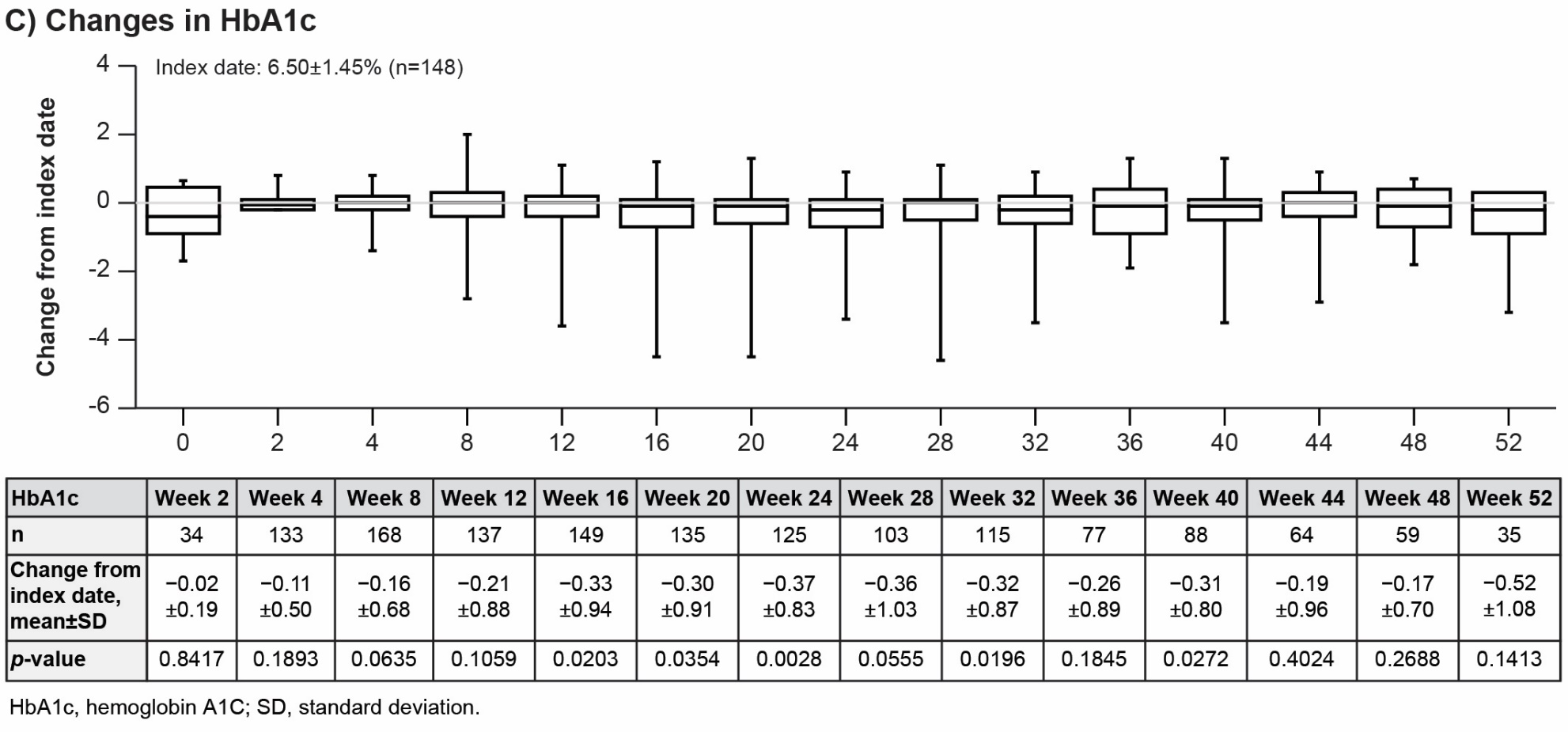
**

**Supplementary Figure 2.** Changes in laboratory measures in eligible population from the initiation of Sac/Val prescriptions (index date). A) Changes in eGFR; B) Changes in potassium; C) Changes in HbA1c.
